# 

**Published:** 1869-07-01

**Authors:** 


### *CONTENTS.*

TRANSLATIONS.

Anatomical and Physiological Observations made upon decapitated subjects. By M. C. Robin .......... 397

REPORTS.

Chicago Medical Society .......... 406

North Iowa Medical Society .......... 408

ORIGINAL COMMUNICATIONS.

Philadelphia Letter .......... 410

MISCELLANY.

The Microscope in Sterility. By J. Marion Sims, M.D .......... 411

Death from Chloroform .......... 421

Contagion of Consumption .......... 424

Carbolic Acid .......... 425

Two Interesting Parasitic Diseases .......... 426

Calabar Bean .......... 427

OBITUARY .......... 428

